# Neurosarcoidosis in a 67-Year-Old Male Without Pulmonary Involvement: A Case Report

**DOI:** 10.7759/cureus.69077

**Published:** 2024-09-10

**Authors:** Muhammad Umar Mian, Hassan Abdullah, Saad Nadeem, Moeed Ahmad, Rashid Siddiqui

**Affiliations:** 1 Internal Medicine, Allama Iqbal Medical College, Lahore, PAK; 2 Internal Medicine, Ittefaq Hospital, Lahore, PAK; 3 Internal Medicine, Lincoln County Hospital, Lincoln, GBR

**Keywords:** altered mental status, chronic granulomatous condition, neurosarcoidosis, non-pulmonary sarcoidosis, sarcoidosis in elderly male

## Abstract

Neurosarcoidosis is a rare and complex form of sarcoidosis that affects the nervous system, presenting significant diagnostic and therapeutic challenges due to its varied neurological symptoms and potential complications. We present a case of a 67-year-old immunocompetent male who presented with altered sensorium, prompting a thorough evaluation. His medical history revealed intermittent low-grade fevers, progressive weight loss, and frailty, rendering him bedridden for over a year. Previous blood tests had ruled out specific etiologies, with normal serum calcium and angiotensin-converting enzyme (ACE) levels. Upon presentation, further diagnostic workup included imaging and laboratory tests. Results showed elevated serum calcium, increased ACE levels, and depressed intact parathyroid hormone levels. MRI of the brain with contrast revealed lobulated mucosal thickening in the right sphenoid sinus and adjacent anterior cavernous sinus. A CT scan of the chest was unremarkable. Additionally, a splenic biopsy revealed hypoechoic foci with neutrophilic, lymphocytic, and histiocytic granulomas. Based on imaging and histopathological findings, the patient was diagnosed with neurosarcoidosis. The patient was treated with prednisolone and methotrexate, leading to a prompt improvement in consciousness and symptoms. Follow-up demonstrated continued improvement and stabilization of his condition. This case highlights the importance of considering neurosarcoidosis in patients with unexplained neurological symptoms and underscores the value of a multidisciplinary approach in managing this challenging condition.

## Introduction

Sarcoidosis is a systemic granulomatous disease characterized by non-caseating granulomas in various organs, most commonly the lungs. While it typically presents with pulmonary symptoms, sarcoidosis can also manifest as isolated extrapulmonary involvement [1]. The etiology of sarcoidosis remains unknown, but it is thought to be triggered by an immune response to factors such as infections or chemicals in genetically predisposed individuals [2]. Neurosarcoidosis, a rare form of extrapulmonary sarcoidosis, affects the central nervous system and is often difficult to diagnose due to its diverse presentation and overlap with other neurological conditions [3]. Although neurosarcoidosis can present with various symptoms, cranial nerve abnormalities are the most common. Only about 1% of individuals with sarcoidosis have isolated neurosarcoidosis without other organ involvement. Diagnostic investigations, including serum levels of angiotensin-converting enzyme (ACE) and calcium, along with MRI of the brain, are crucial for diagnosing neurosarcoidosis [4,5]. This case report highlights the need for a comprehensive evaluation to effectively identify and manage non-pulmonary forms of sarcoidosis. We present a unique case of neurosarcoidosis in a 67-year-old immunocompetent male with an atypical clinical profile.

## Case presentation

A 67-year-old gentleman, normotensive and a known diabetic managed on metformin, presented to us on May 13, 2023 with altered mental status and complaints of intermittent fever for the past year and a half. The fever was not associated with rigors, chills, cough, sputum expectoration, vomiting, diarrhea, bone or joint pains, or any other localizing symptoms. The patient had no additional comorbidities but had experienced substantial weight loss of approximately 45 kg over the course of his illness, leading to a significant decline in functional capacity, with the patient spending most of his day bedridden.

For the past three months, the patient had been undergoing a workup for pyrexia of unknown origin (PUO). Initial tests revealed normocytic normochromic anemia, an erythrocyte sedimentation rate of 50 mm/hour, and mildly deranged liver function tests, with serum alkaline phosphatase at 141 U/L and gamma-glutamyl transferase at 140 U/L. Serum electrolytes were normal, with sodium at 138 mEq/L and potassium at 4 mEq/L. The initial serum calcium level was 9.1 mg/dL. Anti-nuclear antibody titer was negative, and further fever workup showed three negative blood films for malarial parasites and negative brucella antibodies.

Ultrasound of the abdomen revealed hepatosplenomegaly, with the liver measuring 176 mm in the mid-clavicular line, showing fatty infiltration but no focal lesions. The spleen was also enlarged, measuring 161 mm and exhibiting multiple hypoechoic foci with a heterogeneous echotexture. The patient had three negative blood cultures and a negative urine culture, leaving the cause of the fever unexplained.

On current admission, the patient presented with an altered state of consciousness, with a Glasgow Coma Scale score of 10/15. The patient was drowsy but arousable, with a supple neck, equal reactive pupils, a normal cranial nerve exam with no ophthalmoplegia or facial droop, and intact corneal, doll’s eye, gag, and cough reflexes.

Routine laboratory tests revealed hypercalcemia with a corrected calcium level of 14 mg/dL. Renal function tests were normal, with serum blood urea nitrogen at 38 mg/dL and serum creatinine at 0.56 mg/dL. A complete blood count (CBC) showed normocytic anemia with hemoglobin at 9.5 g/dL, a total leukocyte count of 11,400 × 10^9^/L, and a platelet count of 245 × 10^9^/L. Further evaluation of hypercalcemia showed a suppressed fasting parathyroid hormone level of 8.45 pg/mL and an elevated ACE level of 80 U/L. The altered state of consciousness was assessed with a CT head scan and a lumbar puncture, both of which returned normal. CSF analysis showed normal glucose levels of 70 mg/dL, a normal CSF protein level of 24 mg/dL, and only one lymphocyte in the CSF (Table 1).

**Table 1:** Blood and CSF findings ACE: angiotensin-converting enzyme; ALP: alkaline phosphatase; ALT: alanine aminotransferase; BNP: brain natriuretic peptide; CPK: creatine phosphokinase; GGT: gamma-glutamyl transferase; iPTH: intact parathyroid hormone; LDH: lactate dehydrogenase; TIBC: total iron binding capacity

Test	Reference range	Result
Complete blood count		
Hemoglobin	13-17 g/dL	9.5
White blood cells	4-10 × 10^9^/L	11.4
Platelets	150-400 × 10^9^/L	245
Renal function tests		
Blood urea nitrogen	7-18 mg/dL	38
Creatinine	0.5-1.3 mg/dL	0.56
Serum electrolytes		
Sodium	136-146 mEq/L	141
Potassium	3.5-5.0 mEq/L	3.5
Calcium	8.6-10.2 mg/dL	14
Magnesium	1.5-2.0 mg/dL	1.8
Liver function tests		
Bilirubin	0.1-1.20 mg/dL	0.8
ALT	<45 U/L	49
GGT	<59 U/L	221
ALP	50-116 U/L	402
AST	<35 U/L	115
Pro BNP	<125 pg/mL	387.5
CRP	0.8-1 mg/dL	127.8
CPK	25-90 U/L	61
iPTH	15-65 pg/mL	8.45
ACE	8-65 U/L	80
Iron profile		
TIBC	250-400 µg/dL	155
LDH	140-280 U/L	144
Vitamin B12	190-950 pg/mL	>2,000
Reticulocyte count	0.5-2.5%	3.20%
Serum iron	65-175 µg/dL	66
Folate	2.5-20 ng/mL	12.2
CSF analysis		
Microscopy		
RBCs	<1/mm^3^	2
WBCs	0-5/mm^3^	1
Lymphocytes	40-80%	100%
Chemistry		
Glucose	40-70 mg/dL	70
Protein	<40 mg/dL	24
LDH	<40 U/L	16

**Figure 1:**
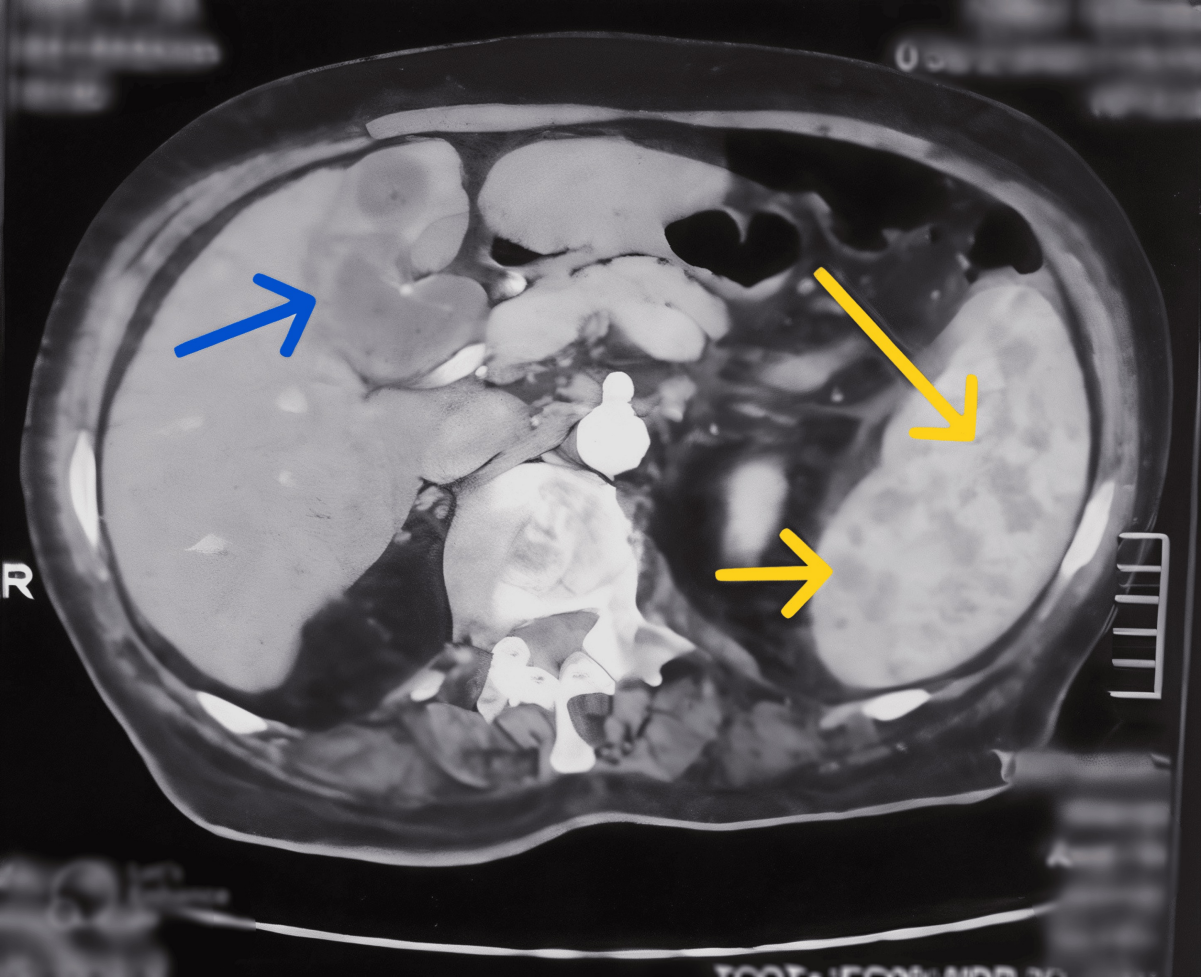
CT scan of the abdomen showing hypoechoic foci in the liver (blue arrow) and spleen (yellow arrows)

With evidence of systemic granulomas on CT abdomen in the liver and spleen (Figure 1), hypercalcemia, elevated ACE levels, and neurological involvement, neurosarcoidosis was considered as a possible diagnosis. An MRI brain with intravenous contrast was performed to further investigate this possibility.

**Figure 2:**
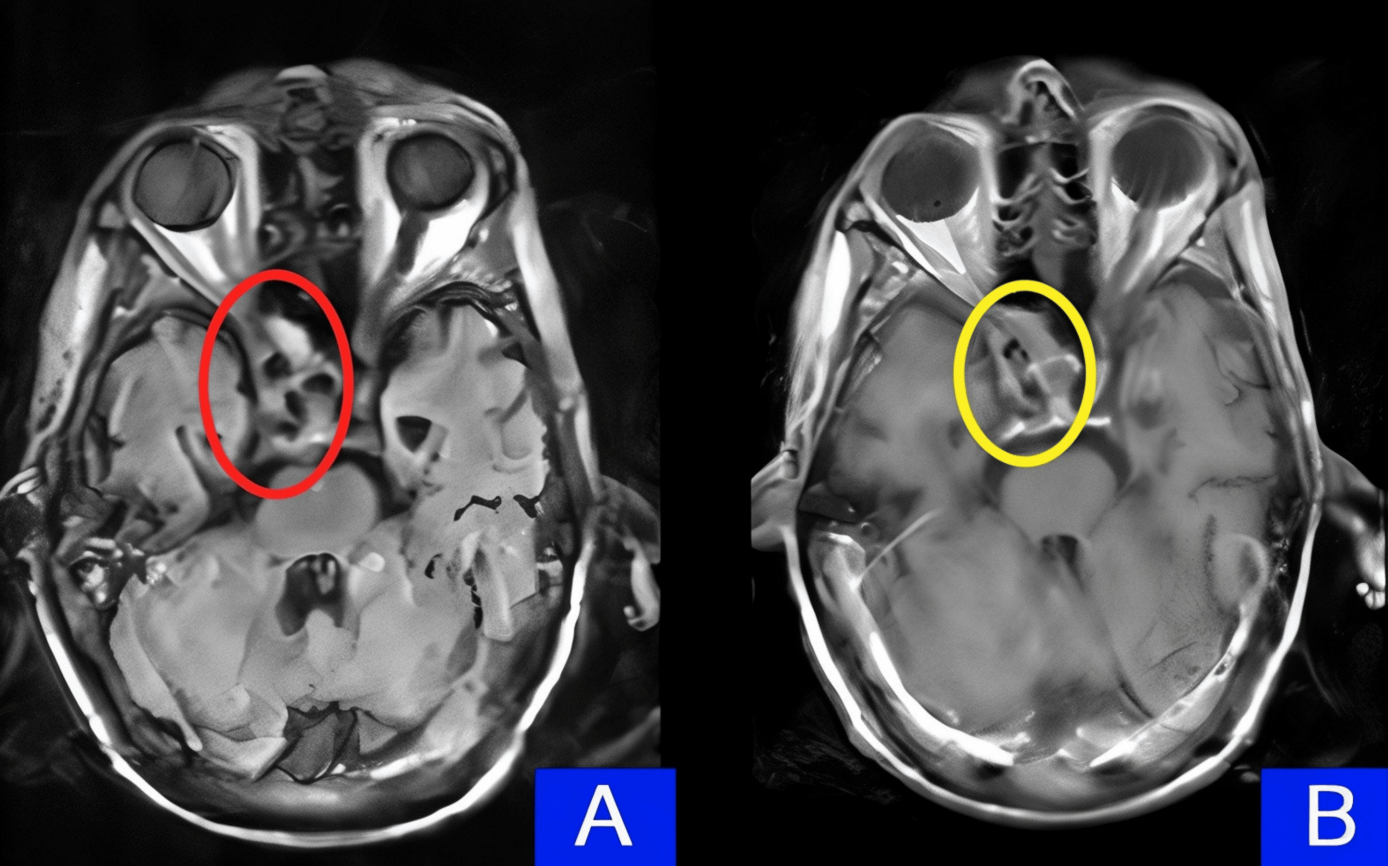
MRI brain: (A) FLAIR sequence showing meningeal signal enhancement in the right anterior cavernous sinus (circled in red). (B) MRI brain without contrast showing pachymeningitis (circled in yellow)

The MRI with contrast revealed lobulated mucosal thickening involving the maxillary sinus and the adjacent anterior part of the cavernous sinus, consistent with pachymeningitis, which is indicative of a granulomatous process such as sarcoidosis (Figure 2).

**Figure 3:**
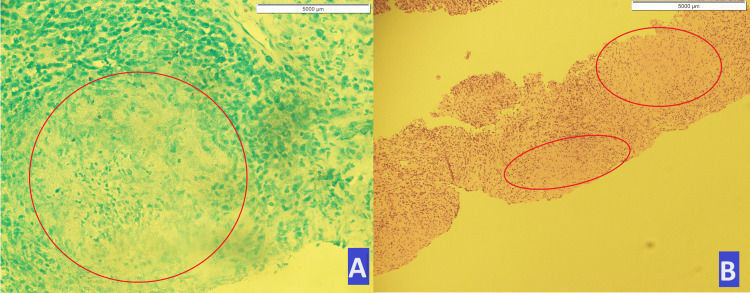
Splenic tissue: (A) Vague, poorly formed, non-caseating granuloma with histiocytes. (B) Multiple poorly formed, non-caseating granulomas with epithelioid cells, neutrophils, and lymphocytes

Subsequently, the patient underwent a trucut splenic biopsy, which revealed a mixed infiltrate of neutrophils and lymphocytes, along with nonspecific histiocytic granulomas (Figure 3).

The patient was initiated on pulsed intravenous methylprednisolone at 1 gram daily for three days, followed by oral prednisolone therapy. He experienced a dramatic recovery with improved consciousness within two days of starting the steroids. The patient was discharged on a daily dose of 30 mg of prednisolone and 10 mg of methotrexate once weekly.

On subsequent follow-up visits, the patient had gained approximately 30 kg, with significant improvement in fatigue, appetite, and resolution of febrile episodes. He reported that the severe fatigue experienced during his illness had markedly improved within weeks, and he was now fully mobile.

The patient is monitored every two months with a CBC, liver function tests, and renal function tests to assess for methotrexate toxicity. He remains stable on 10 mg of prednisolone, with a plan to taper the dose gradually over the next few months.

## Discussion

Neurosarcoidosis is considered one of the uncommon manifestations of sarcoidosis, with a prevalence of only 5-10% of the patients affected by sarcoidosis. The primary disease is characterized by the presence of non-caseating epithelioid granulomas that can be present in any organ of the body, including the lungs, liver, spleen, and brain [6]. However, the lungs are the single most common presenting site, with about 90-95% of the cases being localized to this organ [7].

Sarcoidosis is primarily considered an inflammatory condition, with only some forms of it deemed as autoimmune in nature [3]. The pathophysiology is mainly thought to commence from exposure to an antigen that leads to the formation of a granuloma that subsequently activates the T-cell and macrophage cell lines by the major histocompatibility complex class II (MHC-II). The T-cell lineage primarily includes the CD4-positive T-helper cells. Macrophages and dendritic cells acting as the primary antigen-presenting cells release chemokines and cytokines, including interferon-gamma, tumor necrosis factor alpha (TNFα), and the IL, including IL-2, IL-6, IL-12, IL-15, IL-16, and IL-18 [8].

The most common demographic of neurosarcoidosis is young, African American females. Interestingly, smoking has been associated with a decreased risk of developing sarcoidosis due to unknown reasons. Most cases are seen clustered in the same families, occupations, and locations, inviting further research into their causality [7]. This was also one of the reasons for low suspicion in this case, as our patient had a mismatch of demographics as well as no family history of a similar condition. The exact risk factors and etiology of neurosarcoidosis are still unknown [9]. However, a number of potential causalities have been hypothesized, namely, environmental exposures like beryllium and dust and genetic components, especially ones pertaining to the MHC genes in general and the DR alleles in particular [10].

Sarcoidosis is primarily nonspecific in presentation, with symptoms such as fatigue, malaise, low-grade fevers, and weight loss as common initial findings. Yet, many cases are asymptomatic as well. Sarcoidosis is a frequently neglected cause of PUO. Pulmonary involvement is the single most common presentation with symptoms such as dry cough, chest tightness, dyspnea, and wheezing. The most common extra-thoracic manifestation is cutaneous sarcoidosis, with a prevalence of 20-40%. Erythematous, violaceous, hypo/hyperpigmented papules/plaques, painless subcutaneous nodules in the adipose tissue under the skin, painful nodules called erythema nodosum on the anterior lower extremities, profuse sweating, scarring/non-scarring alopecia, and onycholysis of the nails are the most common cutaneous sarcoidosis manifestations [9]. Ocular sarcoidosis is the second most common extra-thoracic manifestation, presenting as anterior, intermediate, posterior, or panuveitis [11].

Neurosarcoidosis can present as a number of clinical manifestations. Cranial nerves II to VIII can all be involved, with unilateral or bilateral cranial nerve VII (facial nerve) palsy being present in more than 70% of these cases. Orbital and cavernous sinus involvements lead to pain, proptosis, diplopia, and facial sensory impairments. Peripheral neuropathy is present in 10-14% of cases. The neuroendocrine manifestations include panhypopituitarism, diabetes insipidus, and corticoadrenal insufficiencies. Pachymeningitis with dural inflammation or leptomeningitis can both be seen in any part of the cranial cavity, particularly the basal regions [12].

Once suspected, the most common initial diagnostic tests include an ophthalmologic exam, nasal/sinus examination, a chest x-ray, and ACE levels, among other basic first-line investigations such as a CBC, a comprehensive metabolic panel, and other blood and urine tests [6]. MRI brain is the most important imaging diagnostic modality for neurosarcoidosis. It shows positive findings in almost 100% of the patients with neurologic disease [12].

Serum biomarkers are important for ruling out other diseases mimicking sarcoidosis, as they are generally sensitive but not specific for sarcoidosis in particular. An increased CD4/CD8 ratio has a sensitivity between 54% and 80% and a specificity between 59% and 80%. Rituximab targets CD20 on the surface of naïve B cells and memory B cells and has been shown to improve sarcoidosis symptoms. Some chemokines, including CXCL 9, CXCL 10, and CXCL 11, have also been shown to be present in sarcoidosis-induced inflammatory granulomas [13].

CSF analysis is another common diagnostic modality that is used for neurosarcoidosis after a brain MRI. However, leptomeningeal involvement correlates with higher chances of CSF abnormalities pointing towards sarcoidosis, hence aiding in delineating the nonspecific nature of imaging modality utilization alone. CSF analyses can also be used to monitor disease progression and treatment response [14]. CSF analyses commonly present with a high protein count, low glucose, pleocytosis with lymphocytic predominance, and oligoclonal bands raising an important differential diagnosis of neurosarcoidosis versus multiple sclerosis [15]. As discussed above, our patient had atypical CSF findings, contributing to further delays in the correct diagnosis. A biopsy is usually warranted once substantial clinical plus radiological evidence has been obtained. It is usually obtained via the most accessible of the involved locations. These usually include the palpable lymph node, transbronchial, or skin biopsies [15].

The treatment of neurosarcoidosis is largely reactive since it has no reported cure. Spontaneous remissions are possible, but patients usually require long-term, disease-modifying therapies. Exacerbations are tackled by corticosteroid use, which can be tapered off gradually if a favorable response is seen. Immunosuppressant agents like methotrexate, cyclosporine, cyclophosphamide, and TNFα inhibitors like adalimumab have been used with varying success rates. Intravenous immunoglobulins have been shown to have some benefits for patients with neurological involvement. Low-dose radiation therapy is a viable alternative, especially for patients who have been refractory to corticosteroids and other agents. Hydrocephalus can be an uncommon manifestation of neurosarcoidosis, which can be managed by a ventriculoperitoneal shunt. Neurosurgery involving the removal of the space-occupying brain lesion has not shown any benefits [16].

Neurosarcoidosis is a subacute or chronic granulomatous condition that can substantially decrease the quality of life of the affected patients [17]. Prompt diagnosis and management are of the utmost importance when it comes to decreasing the morbidity and mortality associated with this long-term debilitating condition. Many patients go on to carry the burden of this disease for prolonged durations of time, as was the case with our patient. This paper hopes to increase the frequency of clinical suspicion and the subsequent, prompt diagnosis and management of this disease, regardless of the patient demographic.

## Conclusions

Neurosarcoidosis is an uncommon presentation of sarcoidosis, particularly when it occurs without classic pulmonary involvement. Typically, patients present with nonspecific systemic symptoms that are challenging to localize. Often, these patients eventually arrive at emergency departments with acute symptoms such as stroke, meningoencephalopathy, or altered mental status.

In this case, the patient exhibited normal serum calcium and ACE levels over a period of one and a half years, despite the absence of pulmonary symptoms. This highlights that serum calcium and ACE levels are not always reliable for diagnosing sarcoidosis. Clinicians should maintain a high index of suspicion even in atypical cases. Neuroimaging remains the cornerstone of diagnosis, while corticosteroids are crucial for managing acute exacerbations. Long-term treatment options, such as TNFα inhibitors, are effective for many patients. Neurosarcoidosis presents a significant diagnostic challenge, regardless of the presentation. Prompt diagnosis and management are essential for improving long-term prognosis and quality of life for affected patients.
